# 乳腺癌患者再发第二原发性肺癌发病机制及危险因素的研究进展

**DOI:** 10.3779/j.issn.1009-3419.2022.101.46

**Published:** 2022-10-20

**Authors:** 迪 陈, 要来 肖, 铠泽 钟

**Affiliations:** 1 272000 济宁，济宁医学院临床医学院 Clinical College of Jining Medical University, Jining 272000, China; 2 272000 济宁，济宁市第一人民医院呼吸与危重症医学科 Department of Respiratory and Critical Care Medicine, Jining No.1 People's Hospital, Jining 272000, China; 3 272000 济宁，济宁市第一人民医院胸外科 Department of Thoracic Surgery, Jining No.1 People's Hospital, Jining 272000, China

**Keywords:** 乳腺癌, 第二原发性肺癌, 危险因素, Breast cancer, Second primary lung cancer, Risk factors

## Abstract

乳腺癌和肺癌是我国最常见的两种恶性肿瘤。随着乳腺癌诊断和治疗技术的不断提高，乳腺癌患者的生存时间得以延长，乳腺癌患者再发第二原发性肺癌（second primary lung cancer, SPLC）的患者数量不断增加。另外，SPLC的女性患者中最常见的第一原发癌是乳腺癌，且SPLC是此类人群最主要的死亡原因，越来越多的医师开始重视这一临床现象。本文就乳腺癌患者发生SPLC的风险及危险因素进行总结，同时阐述其发病机制，以期为乳腺癌患者的临床管理提供理论依据，尽早实现精准早期干预。

## Abstract (en)

Breast cancer and lung cancer are the two most common malignancies in China. With the continuous improvement of breast cancer diagnosis and treatment technology, the survival time of breast cancer patients has been prolonged, and the number of breast cancer patients with second primary lung cancer (SPLC) has increased. In addition, breast cancer is the most common primary cancer in female patients with SPLC, and SPLC is the main cause of death in this population. More and more physicians pay attention to this clinical phenomenon. This paper summarized the risk and risk factors of SPLC in breast cancer patients, and elaborated its pathogenesis, in order to provide a theoretical basis for the clinical management of breast cancer patients and achieve accurate early intervention as soon as possible.

乳腺癌是全世界和我国最常见的女性恶性肿瘤，新发病例数逐年增加^[1, 2]^。随着乳腺癌早期筛查技术（彩色多普勒超声、钼靶X线等）的逐步普及以及临床诊治水平的不断提高，乳腺癌患者的5年生存率超过85%^[3, 4]^。由于生存时间的延长，乳腺癌患者发生其他原发性恶性肿瘤的可能性增加，第二原发性恶性肿瘤的10年、15年和20年累积发病率分别约为7.43%、14.41%和20.08%^[5]^；另一方面，肺癌因其高发病率和高死亡率对公众健康构成严重威胁，而肺癌作为第二原发性恶性肿瘤的女性患者中最常见的第一原发癌是乳腺癌，占35.1%，且肺癌是此类患者中最主要的死亡原因^[6]^。因此，越来越多的医师开始关注乳腺癌再发第二原发性肺癌（second primary lung cancer, SPLC）的临床现象。

## 1 乳腺癌患者再发第二原发性肺癌（second primary lung cancer, SPLC）的风险

女性乳腺癌患者和非乳腺癌患者的肺癌发病率分别为8.20/10,000人年和5.94/10,000人年^[7]^。乳腺癌患者随访7年-11年、12年-16年分别约有1%和1.7%发生SPLC^[8]^。以往的研究中，乳腺癌是否增加SPLC的发生风险存在不一致结果，有的认为增加了风险，有的认为未增加风险。近期两项*meta*分析^[9, 10]^和一项高质量研究^[7]^显示女性乳腺癌患者发生SPLC的的风险是普通人群的1.18倍-1.34倍，随着随访时间的延长，发生风险似乎没有线性增加。

## 2 乳腺癌患者再发SPLC的发病机制

乳腺癌与肺癌的发生存在某些共同机制，如基因、晚期糖基化终产物受体（receptor for advanced glycation end products, RAGE）、雌激素及烟草等。乳腺癌放疗则通过一些直接和间接作用在一定程度上促进SPLC的发生。对于乳腺癌患者再发SPLC风险增加的内部机制尚缺乏深入研究，需要未来不断补充。

### 2.1 基因与癌症

一些基因异常表达与乳腺癌和肺癌的发生密切相关，如*BRCA*基因（抑癌基因）、*MLH1*/*MSH2*、*p53*和*SH2B3*基因（编码的SH2B3是细胞因子信号传导的关键负调节因子），一定程度上反映了由基因突变所赋予的共同的遗传易感性^[11-14]^。

### 2.2 RAGE与癌症

RAGE是一种多配体跨膜受体，属于免疫球蛋白超家族，由晚期糖基化终产物（advanced glycation end products, *AGEs*/*AEGR*）基因编码。*AGER*基因具有多种遗传变异形式，其中位于*AGER*基因启动子中的-374T＞A（又称rs1800624）、-429T＞C（又称rs1800625）多态性通过影响转录因子位点的结合亲和力增加体外AGER和RAGE的转录活性^[15, 16]^，可能增加乳腺癌和肺癌的发病风险^[17, 18]^。

RAGE的细胞外结构域与多种配体结合，包括AGEs、β-淀粉样蛋白（amyloid β protein, a-β）、S100蛋白家族和溶血磷脂酸（lysophosphatidic acid, LPA）等。配体-RAGE轴可以触发一系列与癌症相关的信号传导。AGEs、a-β通过与其受体RAGE相互作用，激活下游信号通路，增加活性氧（reactive oxygen species, ROS）的产生，导致脱氧核糖核酸（deoxyribonucleic acid, DNA）损伤，增加癌变风险^[19]^。S100蛋白家族中的S100A2、S100A4和S100A14可与细胞内的抑癌基因*p53*相互作用，其异常表达使p53的肿瘤抑制活性丧失^[20-22]^，而S100家族中的S100A6和S100A14则激活RAGE介导的ROS产生^[23, 24]^，从而增加肺癌和乳腺癌的发病风险。LPA-RAGE轴主要通过激活其下游的蛋白激酶B（protein kinase B, PKB）进行信号传导，促进癌基因细胞周期蛋白D1和c-Myc的过度表达，导致不受调节的细胞分裂和细胞增殖以促进肺和乳腺肿瘤的发生^[25]^。

### 2.3 雌激素与癌症

雌激素通过雌激素受体（estrogen receptor, ER）进入细胞内，被细胞色素P450家族的酶（CYP1A1、CYP1B1）羟基化产生2-羟基雌激素（2-hydroxy estrogen, 2-OH-E）、4-羟基雌激素（4-hydroxy estrogen, 4-OH-E）。4-羟基雌二醇（4-hydroxy estradiol, 4-OH-E2）可以损害纺锤体组装检查点（spindle assembly chechpoint, SAC）功能导致基因组不稳定并诱导异常有丝分裂产生致癌作用。此外，4-OH-E2具有儿茶酚结构很容易氧化成亲电子的醌和半醌，可与DNA反应形成脱嘌呤加合物，导致DNA嘌呤位突变，致使乳腺癌和肺癌的发生^[26]^。

### 2.4 烟草与癌症

由于烟草烟雾中存在60余种致癌物，吸烟者患肺癌和乳腺癌的风险明显高于不吸烟者^[27]^，且增加了此类患者的总体死亡率和癌症死亡率^[28]^。另外，烟草产品的燃烧会产生还原糖，与血浆和细胞外基质蛋白发生非酶促反应，从而产生称为AGEs的化合物，从烟草衍生的AGEs可与RAGE结合引发一系列事件，导致慢性炎症和细胞DNA损伤，间接促进癌症的发生^[29]^。

烟草烟雾中的部分致癌物与雌激素存在致癌协调作用，如烟草烟雾中的苯并芘能增加机体产生活性氧和雌激素的有毒代谢物（特别是4-OH-E2，具有致突变和致癌作用），因此女性比男性更容易受到烟草诱发的致癌作用^[30]^。

### 2.5 乳腺癌放疗与SPLC

乳腺癌患者接受放疗过程中不可避免地会发生不同程度的放射性肺损伤（radiation-induced lung injury, RILI），包括早期的放射性肺炎和晚期的放射性肺纤维化。辐射诱导的组织损伤主要包括两种机制，直接DNA损伤和ROS的产生：①直接损伤：DNA或细胞器的损伤触发细胞内信号传导，导致基因表达改变和生长因子释放，可能导致细胞癌变；②ROS的产生：水分子电离可以产生ROS，既可以导致DNA损伤，又可以引起炎性细胞产生细胞因子和趋化因子，导致炎症和慢性氧化应激的恶性循环^[31]^。然而，大量研究^[32, 33]^表明炎症反应与肺癌的发生和进展密切相关。

烟草相关致癌物结合辐射的电离效应也可诱导DNA损伤和肿瘤发生，但其分子机制尚不明确。

## 3 乳腺癌患者再发SPLC的危险因素

乳腺癌合并SPLC患者的总生存期低于单纯肺癌患者^[34]^，并且肺癌是此类人群死亡的主要原因^[35]^。基于此，探索乳腺癌患者再发SPLC的危险因素有望被用于临床的早期预防中。

### 3.1 年龄

数据^[36]^显示乳腺癌患者再发SPLC的中位年龄为50岁-59岁。关于年龄对乳腺癌患者再发原发性肺癌的影响，分组多集中在＜50岁和≥50岁，且相关研究结果有矛盾之处。有研究^[7, 10]^报道，乳腺癌患者诊断年龄＜50岁比≥50岁发生SPLC的风险显著升高。有研究^[37]^显示乳腺癌患者发病年龄与SPLC的发生风险之间存在负相关的趋势，但没有达到统计学意义。相反，Wang等^[9]^对9项研究的1,196,357例乳腺癌患者进行汇总，显示乳腺癌诊断年龄（＜50岁 *vs* ≥50岁）与随后发生肺癌的风险之间不存在显著相关性。因此，需要更多高质量的研究探索并验证这一现象。

### 3.2 时间间隔

乳腺癌患者再发SPLC的中位间隔时间为43个月-60个月^[6, 36, 37]^。Wang等^[8]^进行了大数据分析，纳入620,429例乳腺癌患者，其中6,269例继发了原发性肺癌，两癌之间的中位间隔时间为49个月。为了早诊断早治疗再发的原发性肺癌，应严格进行乳腺癌的随访，特别是乳腺癌诊断后3年-5年的肺部评估。

### 3.3 吸烟

吸烟使女性乳腺癌患者继发原发性肺癌的风险增加3.8倍-9.73倍^[9, 38]^。吸烟与放疗联合暴露可能存在协同作用，诱发新癌症的发生。DiMarzio等^[38]^对10,676例乳腺癌（Ⅰ期-Ⅲ期）患者进行研究，发现吸烟放疗组、不吸烟放疗组、吸烟不放疗组发生SPLC的风险分别是不吸烟不放疗组的4.98倍、1.59倍、3.80倍。如果乳腺癌患者接受治疗时开始戒烟，将使其20年内继发原发性肺癌的风险比不戒烟者降低10.6%，凸显了戒烟的重要性^[39]^。另外，吸烟增加乳腺癌后SPLC的死亡风险。乳腺癌治疗时不化疗和化疗患者中，吸烟者死亡风险分别增加37%和25%^[38]^，但未有研究对SPLC患者进行亚组分析。

### 3.4 ER、孕激素受体（progesterone receptor, PR）和人类表皮生长因子受体-2（human epidermal growth factor receptor 2, HER-2）

与普通人群相比，激素受体阳性[ER阳性和（或）PR阳性]乳腺癌幸存者患原发性肺癌的风险高6%，激素受体阴性（ER阴性和PR阴性）乳腺癌患者高22%^[40]^。ER阴性的患者发生SPLC的可能性高于ER阳性患者。Liu等^[41]^回顾性分析了535,941例女性乳腺癌患者，发现ER阳性患者发生肺癌的可能性低于ER阴性患者（RR=0.93, 95%CI: 0.87-0.99, *P*=0.014）。一部分原因可能得益于乳腺癌的内分泌治疗，因为抗雌激素药物治疗与随后发生肺癌的风险降低有关^[42]^，并且他莫昔芬治疗5年比治疗2年的肺癌发病率显著降低，这种现象可以持续到终止治疗后的10年甚至更久^[43]^。

PR的表达状态也观察到了类似结果，阴性的乳腺癌患者发生肺癌的可能性高于PR阳性患者。*HER-2*基因的表达状态则似乎不影响乳腺癌患者发生后续肺癌的风险^[41]^，但Lin等^[37]^报道*HER2*阳性乳腺癌患者发生同步（诊断时间间隔0个月-6个月）肺癌的风险增加，可能遗传背景在疾病表型中起重要作用。由于缺乏相关的深入研究，可能的内部机制尚不清楚。

### 3.5 放疗

放疗是乳腺癌术后综合治疗的重要手段，可抑制乳腺癌的复发。然而，放疗也是乳腺癌患者再发SPLC的独立危险因素。2021年，四川大学华西医院车国卫教授团队对17项回顾性研究的513,344例乳腺癌患者进行了*meta*分析，表明接受放疗比不接受放疗的患者发生SPLC的风险增加了40%，并进一步证实放疗仅增加同侧肺癌发生的风险（RR=1.27, 95%CI: 1.10-1.45, *P*=0.001）^[9]^，这与我们在临床中观察到的结果一致。

乳腺癌治疗后发生SPLC的风险受放射技术、放射距离、放射剂量的影响。Zhang等^[44]^发现放射距离＞3 cm时，继发性肺癌风险降低；切向野容积调强放射治疗（tangential field intensity modulated radiotherapy, 2F-IMRT）继发性癌症的风险低于多野容积调强放射治疗（multiple field intensity modulated radiotherapy, 6F-IMRT）和容积调强放射治疗（volumetric modulated arc therapy, VMAT）。质子束放疗（proton beam radiotherapy, PBRT）与三维适形放疗（3-dimensional conformal radiation, 3DCRT）、IMRT相比，第二次癌症的发生风险较低^[45, 46]^。在乳腺癌后的肺癌研究中，有证据^[47]^表明随着剂量的增加，风险会增加。随着放射治疗技术的显著进步，放疗对后续肺癌发展的影响可能会降低甚至消失。

有学者^[48-51]^提出了一些降低胸部放疗患者放射性肺损伤发生率的方法，如深吸气呼吸控制技术、大剂量近距离治疗加玻尿酸注射和放射性保护剂（如碳化铌、含硒聚合物药物）。适当考虑采取一些保护性措施，以减少正常肺组织的暴露和放射性肺损伤的发生率。

### 3.6 化疗

大约70%的乳腺癌患者接受术后辅助化疗或新辅助化疗，大多数化疗药物具有致癌作用，如烷基化剂（环磷酰胺）和阿霉素。这些化疗药物不仅可以直接损伤DNA和核糖核酸（ribonucleic acid, RNA），还可以降低癌症患者的免疫功能，导致随后发生其他恶性肿瘤。

然而，多项研究^[5, 9, 52]^发现化疗降低了乳腺癌患者再发SPLC的风险，起到了保护作用。推测这一现象可能有以下原因：①化疗是一种全身抗肿瘤治疗，可以杀死全身的肿瘤细胞；②有一个部分用于乳腺癌和肺癌的化疗药物重叠，如用于乳腺癌化疗的紫杉醇、多西他赛、吉西他滨，对肺癌也有一定的治疗或抑制作用；③与未接受化疗的乳腺癌患者相比，接受化疗的患者更容易发生晚期乳腺癌，预后较差，由于生存期短，发生后续原发癌的风险较低。

### 3.7 其他

其他因素也可能对乳腺癌患者发生原发性肺癌的风险产生影响，如乳腺癌分期、酒精等。Liu等^[41]^发现乳腺癌T4、N1、M1分期是发生SPLC相关的重要危险因素。酒精则与肺癌、乳腺癌的患病率和死亡率呈显著正相关^[53]^。

## 4 总结和展望

随着乳腺癌患者生存期的延长，合并SPLC的患者数量不断增加，这一现象逐渐引起了胸外科和肿瘤科医师的广泛关注。

通过对以上问题的探讨，本文发现：①女性乳腺癌患者再发SPLC的风险显著增加；②机体可能通过基因、配体-RAGE轴、雌激素、烟草、放疗等机制促进SPLC的发生；③应严格进行乳腺癌诊断后的肺部评估，尤其是50岁-59岁和乳腺癌诊断后3年-5年的患者；④吸烟是增加后续原发性肺癌的独立危险因素，戒烟刻不容缓；⑤放疗增加后续原发性肺癌的发生危险，应进行个体化治疗，注意放射技术、放射距离、放射剂量等的选择，并采取一些保护性措施；⑥化疗、ER阳性和PR阳性是保护性因素，符合条件时积极进行化疗和内分泌治疗。

目前对乳腺癌再发SPLC发病机制的研究较局限，未来的研究可以深入全面地阐明这一领域，尽早实现临床的精准早期干预，减少SPLC的发生风险。
